# Supplementary material for: Ytterbium-Doped Lead–Halide Perovskite Nanocrystals: Synthesis, Near-Infrared Emission, and Open-Source Machine Learning Model for Prediction of Optical Properties
Source: Nanomaterials (Basel). 2023 Feb 16;13(4):744. doi: 10.3390/nano13040744 (PMC9958719; doi:10.3390/nano13040744)
Supplement: Supplementary file 1 [file nanomaterials-13-00744-s001.zip › nanomaterials-2192597-supplementary.pdf]

nanomaterials

Supplementary Materials

Ytterbium-Doped Lead–Halide Perovskite Nanocrystals: Synthesis, Near-Infrared Emission, and Open-Source Machine Learning Model for Prediction of Optical Properties

Yuliya A. Timkina [1], Vladislav S. Tuchin [1], Aleksandr P. Litvin [1,2], Elena V. Ushakova [1,*] and Andrey L. Rogach [3]

1 International Research and Education Centre for Physics of Nanostructures, ITMO University, Saint Petersburg 197101, Russia
2 Laboratory of Quantum Processes and Measurements, ITMO University, Saint Petersburg 197101, Russia
3 Department of Materials Science and Engineering & Centre for Functional Photonics (CFP), City University of Hong Kong, Hong Kong SAR 999077, China
* Correspondence: elena.ushakova@itmo.ru

Table S1. Experimental parameters for the synthesis of Yb-doped $CsPbCl_3$ pNCs taken from Refs. [15,23,26,45,46,56–58] (see main text).

Chemical formula	DOI	Pb_mmol	Yb_mmol	OlAm_mL	OlAc_mL	temperature_°C	time_sec
Yb:$CsPbCl_3$	10.1039/c8tc03957g	0.12	0.38	2	2	260	5
Yb:$CsPbCl_3$	10.1021/acs.nanolett.8b05104	0.2	0.08	0.5	1	240	1
Yb:$CsPbCl_3$	10.1021/acs.nanolett.8b01066	0.2	0.16	0.5	1	240	1
Yb:$CsPbCl_3$	10.1021/acs.nanolett.7b04575	0.38	0.19	1	1	240	30
Yb:$CsPbCl_3$:Mn(2.17%)	10.1002/advs.202001317	0.2	0.02	1	0.5	200	10
Yb:$CsPbCl_3$:Mn(1.45%)	10.1002/advs.202001317	0.2	0.04	1	0.5	200	10
Yb:$CsPbCl_3$:Mn(1.3%)	10.1002/advs.202001317	0.2	0.06	1	0.5	200	10
Yb:$CsPbCl_3$:Mn(1.14%)	10.1002/advs.202001317	0.2	0.08	1	0.5	200	10
Yb:$CsPbCl_3$	10.1021/acs.jpcc.9b01296	0.4	0.32	1	2	240	1
Yb:$CsPbCl_3$	10.1021/acsami.1c09421	0.2	0.09	1	3	240	1

Table S2. Comparison of the predicted and experimental quantum yields Yb-doped $CsPbCl_3$ in the NIR spectral region reported in Refs. [15,23,26,45,46,56–58] (see main text).

Chemical formula	DOI	Prediction of NIR QY_%	Experimental NIR QY_%	Absolute difference_%
Yb:$CsPbCl_3$	10.1039/c8tc03957g	124.3	120.1	4.2
Yb:$CsPbCl_3$	10.1021/acs.nanolett.8b05104	132.6	114	18.6
Yb:$CsPbCl_3$	10.1021/acs.nanolett.8b01066	155.7	170	14.3
Yb:$CsPbCl_3$	10.1021/acs.nanolett.7b04575	145.4	142.7	2.7
Yb:$CsPbCl_3$:Mn(2.17%)	10.1002/advs.202001317	55.4	32.5	22.9
Yb:$CsPbCl_3$:Mn(1.45%)	10.1002/advs.202001317	61.2	64.6	3.4
Yb:$CsPbCl_3$:Mn(1.3%)	10.1002/advs.202001317	67	103.3	36.3
Yb:$CsPbCl_3$:Mn(1.14%)	10.1002/advs.202001317	72.8	66.3	6.5
Yb:$CsPbCl_3$	10.1021/acs.jpcc.9b01296	147.3	146	1.3
Yb:$CsPbCl_3$	10.1021/acsami.1c09421	105.7	110	4.3

Table S3. Linear regression coefficients for variables used in the model equation:.

$$\text{NIR PL QY (\%)} = \beta_1 \cdot \text{Pb}_{\text{mmol}} + \beta_2 \cdot \text{Yb}_{\text{mmol}} + \beta_3 \cdot \text{OlAm}_{\text{mL}} + \beta_4 \cdot \text{OlAc}_{\text{mL}} + \beta_5 \cdot \text{temperature}_{°\text{C}} + \beta_6 \cdot \text{time}_{\text{sec}}$$

Coefficient	Variables	Value
β_1	Pb_mmol	−93.47
β_2	Yb_mmol	289.10
β_3	OlAm_mL	−84.18
β_4	OlAc_mL	6.14
β_5	temperature_°C	0.68
β_6	time_sec	1.38
